# Image Quality Analysis and Optical Performance Requirement for Micromirror-Based Lissajous Scanning Displays

**DOI:** 10.3390/s16050675

**Published:** 2016-05-11

**Authors:** Weiqi Du, Gaofei Zhang, Liangchen Ye

**Affiliations:** State Key Laboratory of Precision Measurement Technology and Instruments, Department of Precision Instruments, Tsinghua University, Haidian District, Beijing 100084, China; dwq13@mails.tsinghua.edu.cn (W.D.); yelc08@163.com (L.Y.)

**Keywords:** micromirror, Lissajous scanning display, diamond pixel, resolution, contrast ratio

## Abstract

Micromirror-based scanning displays have been the focus of a variety of applications. Lissajous scanning displays have advantages in terms of power consumption; however, the image quality is not good enough. The main reason for this is the varying size and the contrast ratio of pixels at different positions of the image. In this paper, the Lissajous scanning trajectory is analyzed and a new method based on the diamond pixel is introduced to Lissajous displays. The optical performance of micromirrors is discussed. A display system demonstrator is built, and tests of resolution and contrast ratio are conducted. The test results show that the new Lissajous scanning method can be used in displays by using diamond pixels and image quality remains stable at different positions.

## 1. Introduction

Micromirror-based laser scanning technology has drawn the attention of many researchers for a variety of applications, especially for display systems. The ability of a single MEMS micromirror to deflect a laser with a large angle at high frequency makes it possible to create displays with high brightness and resolution [1,2,3,4]. The benefit of the use of MEMS micromirrors is that they will greatly reduce the volume and power of a system.

In a laser scanning display system, a single modulated laser is scanned simultaneously on two axes. The displays can be divided into two concepts based on the type of scanning used, namely raster scanning displays and Lissajous scanning displays. In raster scanning, the fast axis of the micromirror oscillates at the resonance frequency while the slow axis performs a non-resonant motion in order to scan light line by line. Urey realized a raster scanning display by using an electromagnetically driven 2-D micromirror [5]. The major drawback is that such display requires more power due to the absence of harmonics [6,7,8]. Compared to raster scanning, both axes of the micromirror in Lissajous scanning are driven at resonance so that micromirror can perform a large scan optical angle with a small driving voltage [9]. Scholles realized a display based on Lissajous scanning using a 2-D electrostatically-actuated micromirror [10]. The power consumption of Lissajous scanning displays is much smaller while the scanning trajectory is not normalized. Whatever the type of scanning, it is necessary to study the tradeoff between the characteristics of the scanning mirror and display quality in order to design a suitable micromirror.

For display systems, the major concern is image quality, including resolution, contrast ratio and refresh rate, which are determined by the mechanical angle and scan frequency of the micromirror. In the scanning display, the frame frequency should be larger than 25 Hz in order to ensure consecutive images can be obtained instead of flaring graphs due to the effect of the persistence of vision [11]. Wolter pointed out that a micromirror must be considered as an aperture in the optical path [12]. Besides, for both types of scanning display, image quality can be quantified by the number of resolvable spots it achieves, which is determined by the product of the scanning angle and diameter (*θ × D* product) [13]. Theoretical analysis for raster scanning displays has been done according to this theory, while it has not been done for Lissajous scanning displays.

In a general Lissajous system, a high ratio between the two resonant axes is desired. This requires the slow axis to work resonantly at very low frequencies, near 60 Hz, which makes the system sensitive to vibrations. In order to avoid such problem, Hofmann indicated that Lissajous scanning displays with two high frequencies are more robust, which means the fast axis frequency and the slow axis frequency differ only by the image refresh rate [14]. Roscher calculated the optical performance of micromirrors for Lissajous scanning [15]. The basis is that pixels will be modulated when the light passes through. The drawback is that the actual light is a moving light spot. Such a method does not take the ratio between spot size and pixel into account. The brightness and contrast of pixels will be different. Meanwhile, the pixels are horizontally and vertically arranged, while the scanning light trace is not. The center of a pixel and the light spot do not overlap, which leads to varying sizes and contrast ratios of pixels at different positions in an image. Thus, the present theory for Lissajous scanning display is not precise.

This study aims to resolve the existing problem with Lissajous scanning displays and propose a more accurate formula for the optical requirements of micromirrors. The Lissajous scanning trace with two high frequencies is analyzed first. Then the diamond pixel concept is introduced into Lissajous scanning displays in order to improve the image quality [16]. In addition, the optical performance requirements of micromirrors for Lissajous displays are studied based on the scanning display theory. A display system demonstrator is set up to test the contrast and resolution of the projection images.

## 2. Theoretical Analysis

In this section, a theoretical analysis of the image quality of a Lissajous scanning display is discussed. Lissajous scanning is the compound trace of two orthogonal sine movements as follows:

(1) $${x(t)=A}_{1}\sin(2\pi \;f_{1}t)$$

(2) $${y(t)=A}_{2}\sin(2\pi \;f_{2}t)$$

where f_1_ and f_2_ are the frequencies of the two axis. The frequency difference between f_1_ and f_2_ determines the overall shape and the period of the Lissajous figure in which the trace repeats itself. The figure will be stable when the two frequencies are reducible. As discussed in the former section, the fast axis frequency and the slow axis frequency differ by the image refresh rate. In order to perform this scanning, the frequencies of the two axes should satisfy the following conditions:

(3) $$f_{1}{=2Nf}_{0}$$

(4) $$f_{2}{=2(N-1)f}_{0}$$

where N is a constant, and f_0_ is the greatest common divisor of f_1_ and f_2_.

The period of a Lissajous figure determines the frame frequency of the display while the line density of the scanning trace has a crucial impact on its resolution and contrast ratio. Therefore, a rigorous analysis of these two parameters is conducted.

### 2.1. Period

**Figure 1:**
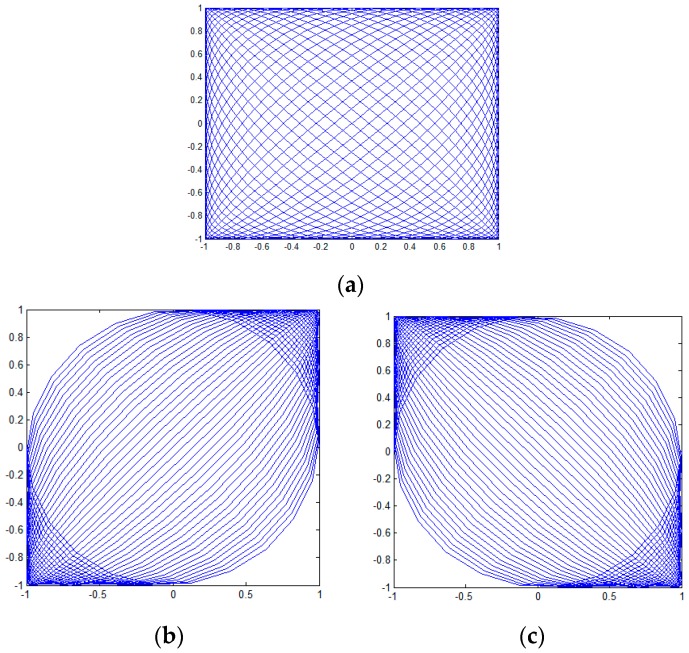
Schematic of a Lissajous scan: (**a**) the full period; (**b**) the first half period (T/4, 3T/4); (**c**) the second half period (T/4, 3T/4).

The scanning period decides the refresh rate of the figure. The full period of the Lissajous trace is presented in Figure 1a. There are intersections during one period of trajectory. The period, T, is calculated by the following equation:

(5) $$T=\frac{1}{f_{0}}$$

Moreover, for the half period (T/4, 3T/4) and (3T/4, 5T/4), the figures are as shown as Figure 1b,c. As demonstrated in Figure 1, most regions can be swept by using half-period Lissajous scanning [17]. Furthermore, the trace can form a tilted raster-like trace without any intersection in the center of the scanning zone. Thus, the mature raster display theory can be used to analyze Lissajous scanning.

### 2.2. Line Density

**Figure 2:**
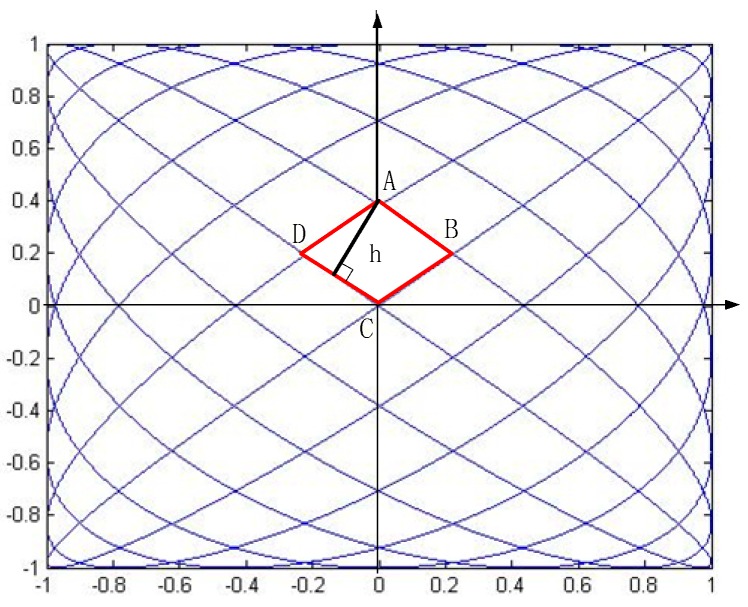
Crossing points of Lissajous figures.

Line density, which is judged by the distance between two adjacent lines, plays a vital role in scanning displays as a measure of the image resolution and contrast ratio. As shown in Figure 2, the Lissajous trace intersects itself at many points. The positions of the crossing points are calculated first [18].

Assuming that the curve traverses the same point at t_1_ and t_2_, the position of two moments will satisfy the following relationship:

(6) $$Z_{1}\left(x\left(t_{1}\right)\text{,y}\left(t_{1}\right)\right){\text{= Z}}_{2}\left(x\left(t_{2}\right)\text{,y}\left(t_{2}\right)\right)$$

It turns out that:

(7) $$t_{1}=k_{x}\Delta ;\;t_{2}=k_{y}\Delta$$

(8) $$\Delta =\frac{1}{8N(2N-1)f_{0}}$$

(9) $$\left\{\begin{array}{l} k_{x}=4N(k_{1}+k_{2})-2k_{1}+2N-1\\ k_{y}=\left|4N(k_{1}-k_{2})-2k_{1}+2N-1\right| \end{array}\right\}$$

or:

(10) $$\left\{\begin{array}{l} k_{x}=4N(k_{1}+k_{2})-2k_{1}+2N\\ k_{y}=\left|4N(k_{2}-k_{1})+2k_{1}+2N\right| \end{array}\right\}$$

Δ is the time constant related to *N* and *f_0_*. *k_x_*, *k_y_*, *k_1_* and *k_2_* are positive integers. It is simple to prove that if 0 ∈ *k_x_*, and *k* ∈ *k_x_*, then (*k* + 1) ∈ k_x_, which also applies to *k_y_*, so *k_x_* and *k_y_* are consecutive positive integers. Hence, the intersection points are passed at the times which are multiples of Δ. Moreover, at the center of a Lissajous figure, the speed of movement of the trace is the greatest and the scanning line is almost straight due to the characteristics of sinusoidal oscillations. Therefore, the maximum spacing between two adjacent scanning lines happens at the center of the scan. As shown in Figure 2, the coordinates of four intersections near the origin are calculated as follows:

(11) $$\begin{array}{l} A\left(0,A_{2}\sin(\frac{\pi }{2N})\right),\;B\left(A_{1}\sin(\frac{\pi }{2(2N-1)}),A_{2}\sin(\frac{\pi }{4N})\right),\\ C\left(0,0\right),D\left(-A_{1}\sin(\frac{\pi }{2(2N-1)}),A_{2}\sin(\frac{\pi }{4N})\right) \end{array}$$

The four points form a diamond-like shape, and the distance between two scan lines, *h*, can be calculated as follows:

(12) $$h=\frac{A_{1}A_{2}\sin(\frac{\pi }{4N-2})\sin(\frac{\pi }{4N})}{\sqrt{{A_{1}}^{2}\sin^{2}(\frac{\pi }{4N-2})+{A_{2}}^{2}\sin^{2}(\frac{\pi }{4N})}}\approx \frac{\pi A_{1}A_{2}}{2N\sqrt{{A_{1}}^{2}+{A_{2}}^{2}}}$$

### 2.3. Method of Display

**Figure 3:**
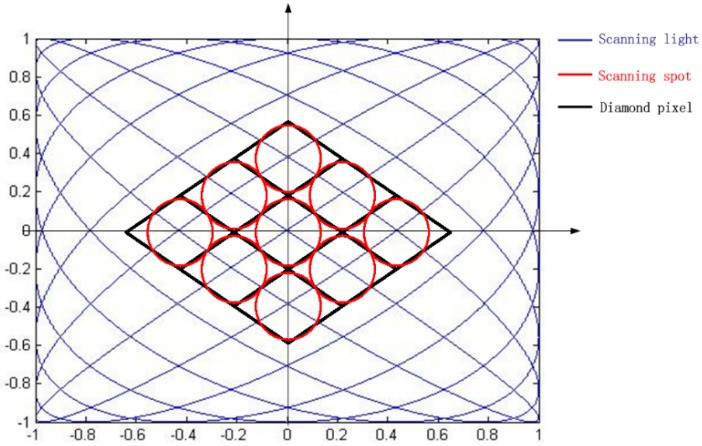
Diamond pixels.

Spot overlap is the ratio of the spot size to pixel size, which means the fill factor of a pixel. In one cycle of Lissajous scanning, the line density is small at the center and big at the rim. As a result, the fill factor of pixels with the same size varies at different positions. To solve this problem, the diamond pixel is adopted in Lissajous scanning displays. The pixel unit of Lissajous scanning displays is set to a diamond-like shape instead of an orthogonal pixel. As shown in Figure 3, the pixel grid is along the scanning trajectory so that the center of the pixel and spot will be overlapped, and the center of the pixel is located at the Lissajous intersection. The sizes of pixels, which can be denoted by the length of the diamond height, *h*, are identified with spot size so that the fill factor at the center of an image is 1.0. However, at the rim of Lissajous scans, the scanning light is not used because adjacent pixels are not distinguishable.

**Figure 4:**
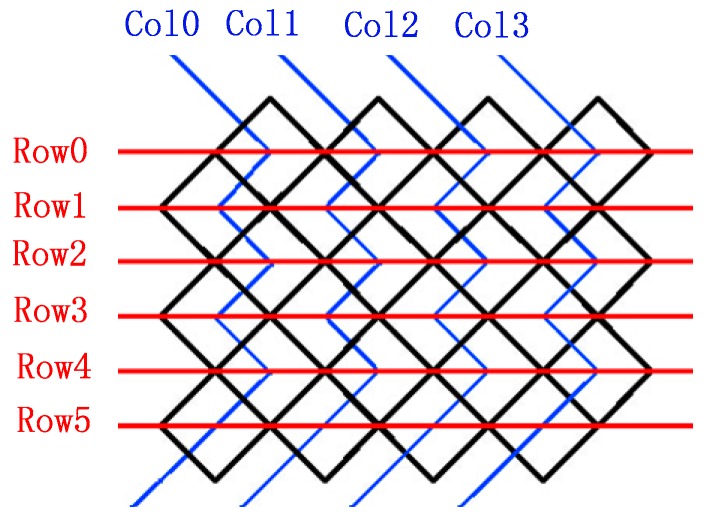
Arrangement mode of diamond pixels.

Diamond pixels and their arrangements were proposed by the TI Corporation [19]. Diamond pixels are rotated 45 degrees compared with traditional pixels, and they are tiled in a zig-zag arrangement, which is shown in Figure 4. The column and row number can be used to evaluate the resolution of a display. For different resolutions, the column and row numbers are different and can be calculated [20].

In the former section, it has been proved that there is no intersection during the half period of Lissajous scanning. The two half periods are the frame periods, *f_r_*, of display in which scanning light takes turns displaying the image. The centers of pixel units are located in the intersections of traces in different half periods so that the center of pixels remain the same during different periods. According to Equation (6), the following equation can be obtained:

(13) $$f_{0}=\frac{f_{r}}{2}$$

## 3. Design Requirements for Micromirrors

### 3.1. θ × D Product

For scanning displays, the micromirror serves as an aperture and diffraction causes a certain divergence of the laser beam that limits the minimum spot size. Here, the spot size is defined as the full-width at 50% irradiance of the Gaussian spot (FWHM) [21]. The resolution can be described by the ratio, *N_R_*, of the maximum scanning angle, *θ*, and the smallest resolvable angle, Δ*θ*, which is related to the scanner diameter, *D*, source wavelength, *λ*, and the shape factor, *a*, which is presented as follows:

(14) $$N_{R}=\frac{\theta_{opt}}{\Delta \theta }=\frac{\theta_{opt}\cdot D}{a\lambda }$$

Urey has introduced the terms *Ksp* and *Kos* [7] in order to illustrate the spot overlap and fill factor, which can also be applied to the Lissajous scanning display in this work. However, the equation is a little different because the scanning trace is tilted. The equation is therefore written as:

(15) $$N_{H}=\frac{\theta_{1}DK_{Sp}K_{os}\cos(\alpha )}{K_{T}a\lambda \cos(\phi )}=\frac{\sqrt{{\theta_{1}}^{2}+{\theta_{2}}^{2}}DK_{Sp}K_{os}\cos(\alpha )}{K_{T}a\lambda }$$

(16) $$N_{V}=\frac{2\theta_{2}DK_{Sp}K_{os}\cos(\alpha )}{K_{T}a\lambda \sin(\phi )}=\frac{2\sqrt{{\theta_{1}}^{2}+{\theta_{2}}^{2}}DK_{Sp}K_{os}\cos(\alpha )}{K_{T}a\lambda }$$

where *N_H_* and *N_V_* are the number of horizontal and vertical diamond pixels, respectively, which can be used to indicate the largest potential resolution of a display system; *θ*_1_ and *θ*_2_ are the horizontal and vertical optical scan angles; *D* is the size of the micromirror; *K_os_* is the overscan constant, which represents the effective scanning area of full scan and is set to 0.707; *K_Sp_* is the spot overlap constant at the center of the image; when *K_sp_* = 1 it means that the pixel size is equal to the spot size; *α* is the feed beam angle; *K_T_* is the beam clipping constant; *a* is the mirror shape factor; *λ* is the wavelength of laser beam; *ϕ* is the slope angle of the scanning beam. At the rim of scanning, the adjacent light beams are not distinguishable so that the scanning light cannot be used. 

### 3.2. Resonant Frequency

Based on the analysis above, because the ratio between FWHM and the pixel size, *h*, is *K_sp_*, the parameter *N* can be obtained by the following equation:

(17) $$N=\frac{\pi \theta_{1}\theta_{2}D}{4K_{T,FWHM}\lambda \sqrt{{\theta_{1}}^{2}+{\theta_{2}}^{2}}}$$

Given the fact that *f_r_* represents the frame frequency of a display, the resonant frequency of two axes can be expressed as follows:

(18) $$f_{1}=\frac{\pi \theta_{1}\theta_{2}Df_{r}}{4K_{T,FWHM}\lambda \sqrt{{\theta_{1}}^{2}+{\theta_{2}}^{2}}}$$

(19) $$f_{2}=(\frac{\pi \theta_{1}\theta_{2}D}{4K_{T,FWHM}\lambda \sqrt{{\theta_{1}}^{2}+{\theta_{2}}^{2}}}-\frac{1}{2})f_{r}$$

Thus, the conclusion is drawn that both frequency and the *θ × D* product have impacts on image quality. According to Equations (15), (16), (18) and (19), the optical performance requirements for micromirrors are as shown in Table 1.

### 3.3. Laser Scanning Time

**Figure 5:**
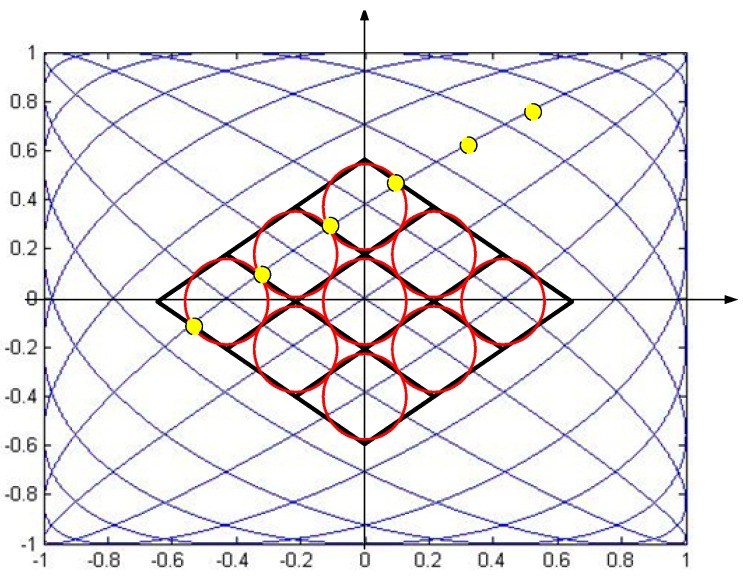
Time array of pixels.

The scanning time for every pixel influences the necessary modulation frequency of the laser. Because the Lissajous figure scans with sinusoidal movement in both axes, the speed of the scanning light reaches its maximum at the center and its minimum at the rim [22]. In order to ensure the size of every pixel remains the same, the actual duration of the light beam at every diamond pixel is calculated, as shown in Figure 5.

The minimum pixel time at the center and the corresponding modulation frequency of the laser for images with different resolutions are shown in Table 2.

## 4. Experiments and Results

A display system was built to realize a projecting display and verify the theory. The resolution and contrast ratio of the system have been tested. All the test pattern photos were taken using a shutter speed of one scanning period.

### 4.1. Demonstrator Setup

**Figure 6:**
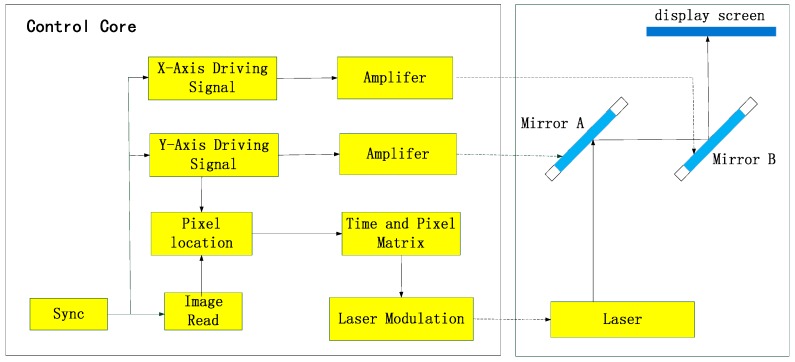
Diagram of the display system.

The diagram of the display system, shown as Figure 6, comprises a modulated laser, two scanning micromirrors, a display screen and a control core circuit. The control core is based on a digital I/O device with 100 KS/s analog output, which is used to generate the pixel matrix of the image. Meanwhile, the sinusoidal driving signals for two micromirrors and modulating signal for the laser are outputs. The driving signals are loaded on the micromirror after amplifying. Besides, the modulating signal ranges from 0~2 V in order to achieve a display with 256 level grayscale.

The performance of the two scanning micromirrors used in system is shown in Table 3. According to the analysis in the previous section, the best resolution this system can achieve is nearly 300 × 300. The scanning period is 5 Hz.

The program is written in Labview and the main function is calculating the position of scanning spots and producing the modulation signal for the laser. The processing steps of the program are as follows:

(1) Read image and generate the pixel matrix of the image (x_i_, y_i_, g_i_). x_i_ and y_i_ are the horizontal and vertical pixels, and g_i_ is grayscale.
(2) Calculate the pixel time based on the scanning frequency of the two axes and generate the time-pixel array (t_i_, g_i_).
(3) Read the first number of time array, t_i_, and accumulate the processing time, t, using the system clock. If (t < t_i_), then the output for laser modulation signal is M × g_i_/256, where M is the max of modulation voltage; otherwise, read the next number in the time array, t_i + 1_, and repeat step 3.
(4) Continue with step 1 after a frame period.

### 4.2. Parameter Test

**Figure 7:**
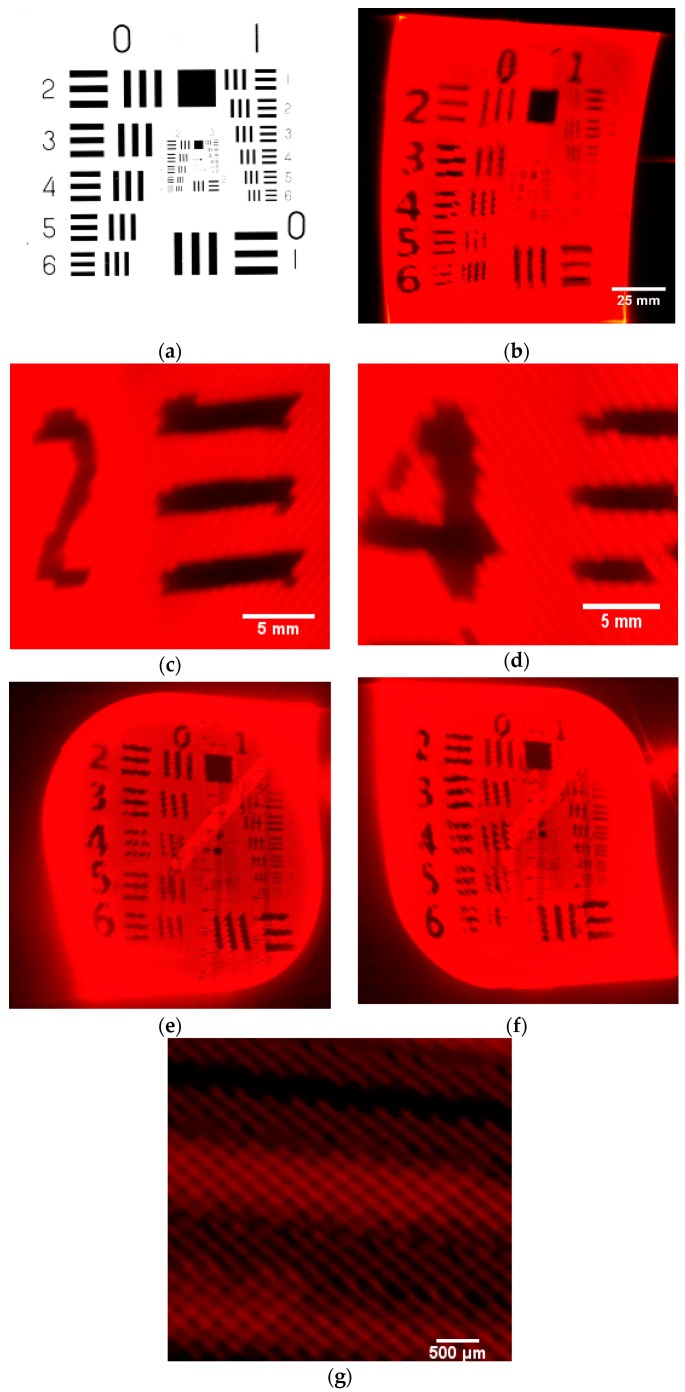
Projection result. (**a**) Test pattern image; (**b**) Projected image; (**c**) Rim of image; (**d**) Center of image; (**e**) Test pattern in the first scanning period; (**f**) Test pattern in the second scanning period; (**g**) Detailed drawing of the diamond pixels.

The resolution test is processed by projecting a test pattern on the screen. Figure 7 shows the 256 × 256 test pattern. The projection of the test pattern is conducted by using a slow shutter speed (1/5th of a second) so that the image of one scanning period is obtained. As shown in Figure 7b, the figure is formed by an array of diamond pixels, and the display effect at different positions of the image is basically the same. However, the projected image does not totally match the original image, and some resolution is lost. The reason for this is that the shape and size of the laser spot that is reflected by two micromirrors does not agree well with the theoretical result. Figure 7e,f show the projection image in two half scanning periods. It is proved that the pattern can be projected by using an effective scanning area in a half scanning period of the Lissajous trajectory. The phase drift of the micromirror oscillation causes the difference between two projected images.

**Figure 8:**
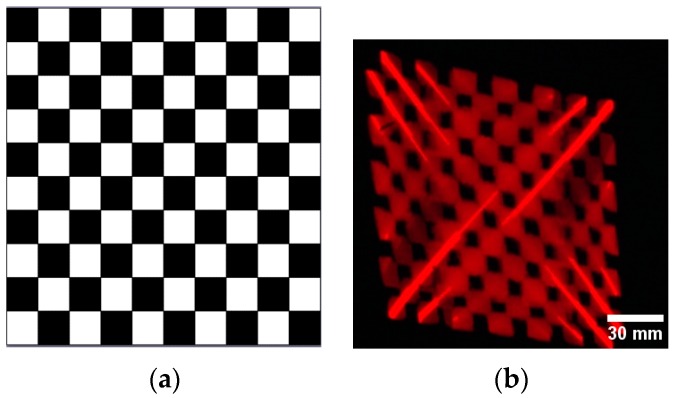
Projection result (**a**) Contrast ratio test image; (**b**) projecting image.

**Figure 9:**
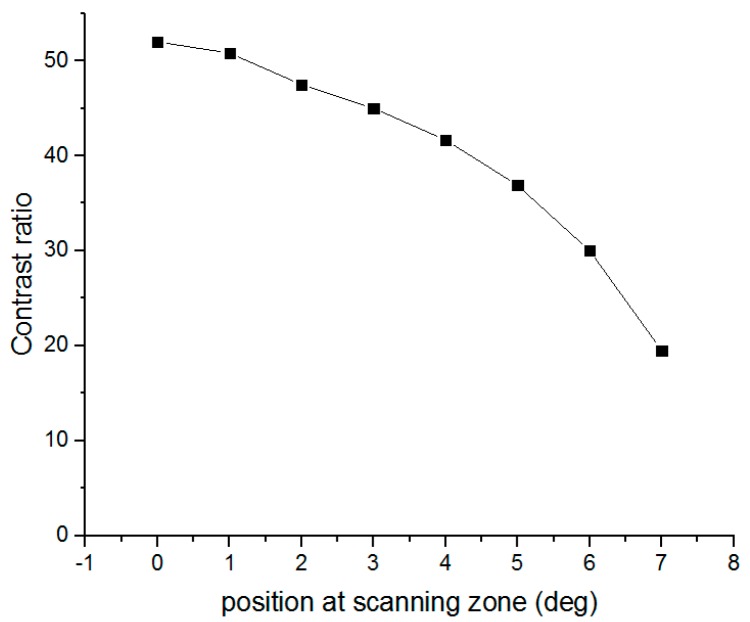
Graph of contrast ratio *vs.* position in the scanning zone.

The system contrast ratio was also tested based on a light meter [23]. Figure 8 is the projection result of the contrast test with five black and white squares per line [24]. The photometer is set to different positions on the image in order to obtain the light intensity, and then the contrast ratio distribution is obtained. The relationship between contrast ratio and position is demonstrated in Figure 9. As presented, the contrast ratio at the center is relatively high, while at the rim of image, it is 38% of max contrast and the figure becomes undistinguishable. Thus, the rim of the Lissajous scanning trace cannot be used in the display. In this paper, the overscan constant is 0.707 corresponding to a contrast of 36.8. Therefore, the contrast ratio decreases by 26.4% when moving from the center to the rim of the scanning zone.

## 5. Conclusions

In this paper a new method for realizing Lissajous scanning displays is presented and diamond pixels are adopted in Lissajous scanning displays. Then the Lissajous trace is analyzed and the optical performance of micromirrors is studied based on image quality. Besides, a demonstrator of the proposed display system is also established, and resolution and contrast ratio tests are conducted. The results show that the new Lissajous scanning method can be applied to displays with the help of diamond pixels. The resolution and contrast remain stable at different scanning positions. However, the resolution of the actual projected image does not yet match the theoretically possible result. A potential reason which explains such results is that the shape and size of a laser spot reflected by two micromirrors is not stable. The display system will be improved for high-definition images. In addition, a single 2-dimensional micromirror with angle feedback for Lissajous scanning displays will be further studied.

## Figures and Tables

**Table 1:** The optical performance requirements for micromirrors.

Requirement	VGA	SVGA	XGA	SXGA	UXGA
Resolution	640 × 480	800 × 600	1024 × 768	1280 × 1024	1600 × 1200
f_r_	60	60	60	60	60
N_H_	453	566	725	906	1132
N_V_	679	849	1087	1449	1698
K_sp_	1	1	1	1	1
K_os_	0.707	0.707	0.707	0.707	0.707
K_T_	1.134	1.134	1.134	1.134	1.134
a	1.22	1.22	1.22	1.22	1.22
λ(μm)	0.635	0.635	0.635	0.635	0.635
θ_1_·D (deg·mm)	27.2	33.6	42.4	52.318	66.4
θ_2_·D (deg·mm)	20.4	25.2	31.8	41.854	49.8
N	313	386	487	626	763
f_1_	18,780	23,160	29,220	37,560	45,780
f_2_	18,720	23,100	29,160	37,500	45,720

**Table 2:** Minimum pixel time and modulation frequency of the laser.

Resolution	VGA	SVGA	XGA	SXGA	UXGA
Minimum pixel time (ns)	25	16	10.5	6.5	4
Modulation frequency (MHz)	40	62.5	96	154	250

**Table 3:** The performance of two micromirrors.

Feature	Mirror A	Mirror B
Frequency (Hz)	790	785
Optical scanning angle (°)	14	14
Mirror size (mm)	2 mm	2 mm
